# Proximal and distal determinants of stressful work: framework and analysis of retrospective European data

**DOI:** 10.1186/1471-2458-14-849

**Published:** 2014-08-15

**Authors:** Morten Wahrendorf, Johannes Siegrist

**Affiliations:** Centre for Health and Society, Institute for Medical Sociology, University of Düsseldorf, 40225 Düsseldorf, Germany; Senior Professorship on Work Stress Research, Faculty of Medicine, University of Duesseldorf, 40225 Düsseldorf, Germany

## Abstract

**Background:**

While robust evidence on associations of stressful work with health exists, less research is available on determinants of stressful work in terms of respondents' characteristics (proximal factors) and in terms of national labour market policies (distal factors). In this article we analyse proximal (childhood circumstances and labour market disadvantage) and distal determinants (national compensation and integration policies) of stressful work in a comprehensive framework.

**Methods:**

We use data from the third wave of the Survey of Health, Ageing and Retirement in Europe (SHARE), with retrospective information on individual life courses collected among 11181 retired men and women in 13 European countries (2008–2009). To test our hypotheses we estimate multilevel regression models.

**Results:**

Results show that stressful work is related to disadvantaged circumstances during childhood. To some extent this association is explained by labour market disadvantage during adulthood. Additionally, well developed labour market integration policies are related to lower overall levels of stressful work at national level.

**Conclusion:**

This analysis provides first evidence of important determinants of stressful work, both in terms of pre-employment conditions (childhood circumstances) and in terms of contextual macro-social policies.

**Electronic supplementary material:**

The online version of this article (doi:10.1186/1471-2458-14-849) contains supplementary material, which is available to authorized users.

## Background

Occupational health research has established solid evidence on the impact of adverse physical and psychosocial working conditions on health, mainly based on epidemiological cohort studies [1–3]. Results of this research are instrumental in terms of scientific innovations, but also in terms of utility as they can instruct stakeholders to develop measures of promoting healthy work [4]. However, these studies usually focus on populations that were already participating in the labour market at the time when investigations started. As a consequence, processes of selection into paid work and their impact on the quality of work and employment received less attention. To some extent, this shortcoming was overcome with the advent of birth cohort studies and longitudinal investigations of adolescent cohorts. These studies demonstrate that adversity in early life, and specifically disadvantaged childhood circumstances, exert negative effects on employment opportunities and quality of work in early adulthood [5–9]. In addition, disadvantaged childhood circumstances were related to reduced health in midlife, in terms of elevated cardiovascular risk. This effect was partly mediated by exposure to stressful working conditions in early stages of occupational life [10, 11]. Yet, due to a relatively short observation period of a majority of birth cohort studies that were initiated in the second half of the last century, there is a lack of knowledge about longer-term effects of adversity in early life on later stages of people’s occupational careers, and specifically on the quality of their main job held until retirement. Is it reasonable to assume that stressful work experienced in the main job of people’s occupational trajectory can be traced back, to some extent, to adverse childhood conditions?

A second limitation of knowledge about determinants of stressful work in people’s occupational careers concerns the potential impact exerted by more distal conditions of national labour and social policies that aim at reducing precarious and unhealthy employment and working conditions. Preliminary evidence indicates that the average level of stressful work among employees of a country is closely associated with the extent to which such policies are implemented. In particular, in countries with well-established active labour market policies lower average levels of stressful work were observed, compared to those in countries with less well developed policies [12, 13]. These findings suggest that such distal contextual factors need to be taken into account in a comprehensive analysis of determinants of stressful work [14–16]. Yet, a more comprehensive assessment of such policies is necessary [17]. Among these contextual factors, two types of labour and social policies are of special interest, protective policies that offer social provision to deprived or disabled people through compensation, and integrative policies that promote return to work and maintenance of jobs [18].

In this contribution we set out to address these two shortcomings of current research on determinants of stressful work by linking proximal (i.e. early life adversity) with distal (i.e. national policies) factors within a conceptual framework and to provide an empirical test of these links. At the proximal level of individual life courses, stressful work is thought to result in part from an increased vulnerability of workers who were deprived from those material and psychosocial resources during their childhood that are critical for successful cognitive, emotional, and social development of core capabilities and coping skills [19, 20]. This early disadvantage may aggravate their access to the labour market and the acquisition of jobs with good or reasonable quality. Jobs with poor quality confer a high level of stress which in turn affects working people with deficient coping resources in a particularly strong way [19].

In addition, the level of stressful work is influenced by contextual conditions at distant level. As mentioned, specific national labour and social policies are thought to protect workers from exposure to fierce market forces, thereby mitigating the severity of stress at work. Yet, in the absence of such protective and integrative policies vulnerable workers may suffer from further aggravation of their amount of work-related stress. In conclusion, a comprehensive analysis that includes proximal and distal determinants allows for a more accurate assessment of the burden of stressful work than is the case in a majority of prevailing studies that are characterized by a less extensive scope of analysis.

Here, we test this conceptual framework by analysing data from the Survey of Health, Ageing, and Retirement in Europe (SHARE) [21]. This survey provides a detailed retrospective assessment of respondents’ previous life [22], thus extending the time window from participants’ current situation and recent past to previous stages of their life course, including childhood conditions and information on entire employment histories (see Methods for details). Based on these data we analyse three hypotheses. First, we assume a dose – response relationship between the degree of childhood adversity and the degree of stressful work experienced later on (hypothesis 1). Second, we assume that this association is partly mediated by a disadvantaged access to the labour market (hypothesis 2). Our third hypothesis relates to the contextual factors. Given the fact that SHARE provides data on national policies from 13 European countries we test the assumption that the average level of stressful work among participants in a country is closely related to the extent to which protective and integrative policies are implemented at national level. Less developed policies go along with higher average levels of work-related stress (hypothesis 3).

## Methods

### Data sources

We used third wave data from the Survey of Health, Ageing and Retirement in Europe (SHARE) collected in 2008–2009. This wave (termed SHARELIFE) is a separate retrospective survey collecting details on participants’ life course. It includes details on previous working careers and childhood conditions [23]. Retrospective data are collected with the lifegrid approach, where recall and timing of major information is supported by a graphical representation of respondent’s life which is filled during the interview. This method was first developed as a self-completion questionnaire [24], and subsequently transformed into a Computer Assisted Personal Interviews (CAPI) by the UK National Centre for Social Research [25]. This latter method was adopted for SHARELIFE [23]. Despite obvious limitations this retrospective assessment offers several advantages. First, it represents an economic way of collecting longitudinal information. Second, it guarantees comparable information referring to different time points in respondents’ life. Furthermore, validation studies revealed high accuracy of recalled information, in particular when asking about socio-demographic conditions [26, 27] and employment histories [28, 29].

For the present analyses, information is available from 13 European countries, ranging from Scandinavia (Denmark and Sweden), Western Europe (Austria, France, Germany, Switzerland, Belgium and the Netherlands), to Mediterranean countries (Spain, Italy and Greece) and to two Eastern European transition countries (Czech Republic and Poland). More details about SHARE are available online (http://www.share-project.org).

### Subjects

In all countries, the sample selection was based on probability household samples where all people plus their partners were interviewed. In total, 26.836 participants were interviewed at wave 3. In the following analyses we included all people who already left the labour market at the time of wave three, provided they documented an employment history of at least 5 years. Restricting the sample to people who left the labour market enabled us to compare employment careers over the whole life course. Importantly, we also excluded respondents older than 80 years as the time since last employment was considered too long for accurate retrospective assessment. This restriction reduced a potential sample bias as people over 80 years may have had more favourable working conditions and related increased survival probability. We also excluded respondents with documented difficulties of answering the lifegrid questionnaire (about 4% of the total sample). These restrictions resulted in a final sample with full available data of 5552 men and 5629 women (N = 11181), born between 1928 and 1959.

### Measures

#### Stressful work

Our measure of stressful work represents a sum score of items derived from two scales. With the first scale respondents were asked to assess in retrospect the degree of adversity experienced in the main job of their occupational career (11 items), with a mean length of 24.5 years in our sample. Each item refers to a core dimension of a stressful work environment, as proposed by the demand-control- support model [30] and the effort-reward imbalance model [31]. The second scale contained 5 items where respondents had to evaluate their overall satisfaction with their entire working career, including its potential impact on their health. For both scales an identical response format was applied (4 categories ranging from ‘strongly agree’ (value 0) to ‘strongly disagree’ (value 3)). When necessary, items were recoded to achieve uniform coding (higher values indicating more stress at work). Cronbach’s alphas were 0.73 (11 items) and 0.63 (5 items) respectively. For the analyses, we constructed a sum score of stressful work with values ranging from 0 (no stress) to 48 (high stress). In addition, we created a binary indicator to identify a critically elevated level of stressful work. To this end, conditions were classified as stressful if respondents reported high work stress (e.g. “agree” or “strongly agree”) for at least half of the items of the two scales (i.e. 8 out of 16 items). All items are presented in a supplementary table in the Additional file 1: Table S1.

#### Childhood circumstances

To measure this variable we combined four binary indicators of disadvantaged childhood circumstances into one index. All indicators refer to respondents’ conditions at age 10 and were used in previous studies of long-term effects of childhood adversity on health in later life [32–35]. First, we included an indicator of occupational position of the main breadwinner at respondents’ age 10, using ten main occupational groups of the International Standard Classification of Occupations (ISCO). These groups were re-classified according to the different skill-levels, representing the broad hierarchical structure of ISCO, which we regrouped into low (1st and 2nd skill level) and high (3rd and 4th skill level) occupational positions [36]. Second, we used a measure of overcrowding by combining information on number of people living in the household with number of available rooms (excluding kitchen, bathrooms and hallways). Following previous studies, overcrowding was coded in all cases where more than one person per room lived in the household [34]. Third, the reported number of books at home was used, and we created the category ‘less than 10 books’ as an indicator of childhood adversity [33]. Finally, we measured housing quality and defined poor quality in the absence of any of the following characteristics: fixed bath, cold running water supply, hot running water supply, inside toilet and central heating [35]. Based on this information, five levels of disadvantaged childhood circumstances were defined ranging from “most advantaged” to “most disadvantaged”.

#### Labour market disadvantage

From detailed information on individual employment histories available in SHARELIFE we developed an index of labour market disadvantage, based on the following four items. The first item asked whether an involuntary job loss occurred as a consequence of being laid off. With the second item involuntary job loss due to plant closure was assessed. Third, we measured the occupational position in respondents’ main job, again based on the ISCO classification (which we regrouped into two categories ‘low and high occupational position’ as described above). With the fourth item an episode of unemployment lasting at least 6 months was registered. By combining these four items, we defined an index with five levels of labour market disadvantage, ranging from “none”, “mild”, “moderate”, “severe” to “very severe” disadvantage. A sample description and overview of all individual variables is presented in Table 1.

**Table 1:** **Sample description: percentage and frequencies (N) or mean scores and standard deviation (SD); (N = 11181)**

***Variables***	***Categories or range***	***% or (mean)***	***N or (SD)***
*Sex*	male	49.7	5552
	female	50.3	5629
*Mean age*	50 – 80	(67.7)	(6.8)
*Retirement age*	before 55	33.3	3718
	55 – 59	27.1	3026
	60 or older	39.7	4437
*Periods of disability*	none	76.9	8549
	one	16.0	1785
	two or more	7.2	802
*Job absence due to disability*	yes	8.1	910
	no	91.9	10271
*Stressful work*	yes	15.9	1775
	no	84.1	9406
*Mean score stressful work*	0 – 48	(19.1)	(6.8)
*Childhood circumstances*	most advantaged	4.7	522
	advantaged	16.2	1809
	neutral	32.0	3578
	disadvantaged	26.3	2944
	most disadvantaged	20.8	2328
*Labour market disadvantage*	none	19.7	2204
	mild	63.1	7055
	moderate	14.0	1567
	severe	2.8	315
	very severe	0.4	40

#### Policy indicators

We used two indices, developed by OECD, that assess two relevant dimensions of labour market policies in case of threats to employment, in particular due to disability, i.e. compensation policies and integration policies [37, 38]. Whilst the compensation (or protection) index is thought to reflect the generosity and accessibility of benefit programmes for the workforce in case of disability, the integration index measures public labour market programs that aim to re-integrate groups of individuals in case of disability. Technically, each index is constructed by the availability and quality of ten policy programmes (e.g.: “sickness benefit level” or “vocational rehabilitation programmes”). These programmes were evaluated by experts for each country separately on a score ranging from 0 to 5 for three years, 1985, 2000 and 2007 [37, 38].

For the analyses, we computed a country mean score for each indicator using data from these three years (with except of Greece and Czech Republic where values were only available for 2007). This temporal extension of information on policy indicators enabled us to relate contextual data to information on stressful work and labour market disadvantage reported during this period. For each country, a respective index varies from a score of zero (poorest policy) to a score of 50 (best policy) (see Additional file 1: Table S2 and [38]).

#### Additional measures

In addition to age, sex, age of retirement, we included two measures of disability, mainly as control variables in multivariate analyses. First, we account for actual disabilities over the life course and include the number of periods (lasting longer than one year) the respondent reported to be “ill or disabled” (regrouped into ‘none’ , ‘one’ , and ‘two or more’). Second, we included a binary indicator measuring whether working life was interrupted due to disability (temporary leave of absence from a job for 6 months or more because of ill health or disability).

### Analyses

**Figure 1:**
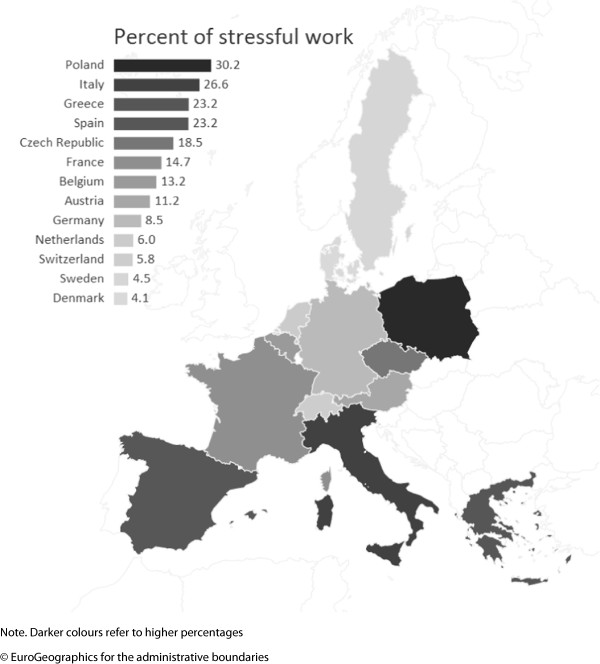
**Percent of stressful work across Europe among older men and women (N = 11181).**

**Figure 2:**
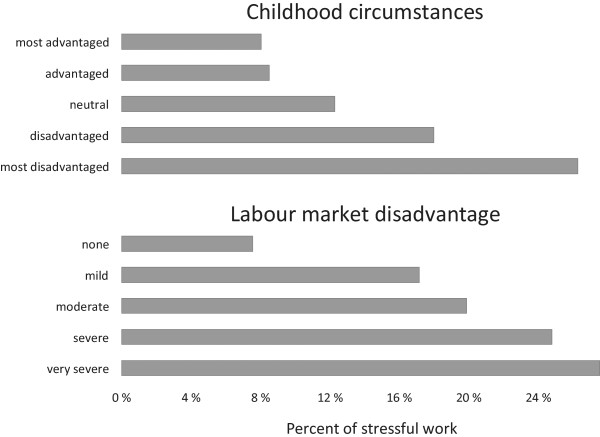
**Percent of stressful work by levels of childhood poverty and labour market disadvantage among older men and women (N = 11181).**

Following a sample description (Table 1), we present descriptive findings for stressful work in respondents’ previous working careers. To allow a detailed inspection of the association of proximate factors with stressful work, the indices of childhood circumstances and labour market disadvantage were included as categorical variables. Country variations are additionally visualized using a geographical map of Europe, where each country is coloured according to existing percentages of critically elevated levels of stressful work (darker colour for higher percentages, Figure 1). In addition, Figure 1 includes a sorted bar chart presenting the percentage of people with stressful work for each country. In Figure 2 we display percentages of stressful work by childhood adversity and labour market disadvantage. Next, to explore associations between distal factors, the two policy indices, and stressful work, two scatterplots are displayed - one for each policy index. Importantly, to account for population compositions and its effect on work stress (e.g. more jobs in lower occupational positions in a country, and thus, more exposure to stressful work), mean scores of stressful work are adjusted for age, gender and all individual characteristics described above.

To test our core research questions, we then estimate a series of multilevel linear models (random intercept only) using the sum score of stressful work as dependent variable with individuals (level 1) nested in countries (level 2) [39]. Using multilevel modelling allows for accurate adjustment for country affiliation when studying effect sizes of proximal determinants because the constant is allowed to vary across countries. Furthermore, variations of stressful work can be studied at each level separately (within- and between-country variations). In addition, likelihood ratio tests were performed comparing the multilevel models to conventional linear regression models (with country dummies), and these tests revealed better model fits in all cases. In sum, we estimate seven different models: The first model contains a constant term only and quantifies the amount of variation of stressful work at each level (Empty Model). Next, we study the role of the proximal determinants and present results of three models, one for childhood circumstances (Model 1), a second one for labour market disadvantage (Model 2), and a third one combining all proximal predictors (Model 3). Model 4 and 5 each includes one of the distal variables (national policy indices), and a final model includes all variables simultaneously (Full Model). The results presented in Table 2 are given as estimated regression coefficients, together with standard errors and level of statistical significance. For each model the log likelihood, the AIC (Akaike Information Criterion) and the BIC (Bayesian Information Criterion) statistics are indicated (lower values represent better model fit in case of AIC and BIC [40]), and the proportional reduction of variance explained at each level (R^2^1, R^2^2) is reported [41]. These latter measures are important because they allow us to quantify the extent to which variations of stressful work at the country-level can be explained by the two policy indices. All analyses were conducted with STATA. Geographical data come from Eurostat, and D3 was used for map projection and data visualization [42].

**Table 2:** **Multilevel estimates for stressful work: Regression coefficients (b) and standard errors (SE) (N = 11181)**

Model		Empty Model		Model 1		Model 2		Model 3		Model 4		Model 5		Full Model	
		b	(SE)	b	(SE)	b	(SE)	b	(SE)	b	(SE)	b	(SE)	b	(SE)
*Fixed parameters*															
*Sex*	female (ref.)														
	male			0.34**	(0.12)	0.54***	(0.12)	0.45***	(0.12)	0.45***	(0.12)	0.45***	(0.12)	0.45***	(0.12)
*Age*				-0.08***	(0.01)	-0.05***	(0.01)	-0.07***	(0.01)	-0.07***	(0.01)	-0.07***	(0.01)	-0.07***	(0.01)
*Retirement age*	before 55 (ref.)														
	55 – 59			-0.35*	(0.16)	-0.3	(0.16)	-0.28	(0.16)	-0.28	(0.16)	-0.27	(0.16)	-0.28	(0.16)
	60 or older			-0.52**	(0.16)	-0.41*	(0.16)	-0.35*	(0.16)	-0.35*	(0.16)	-0.34*	(0.16)	-0.34*	(0.16)
*Periods of disability*	none (ref.)														
	one			1.36***	(0.16)	1.40***	(0.16)	1.35***	(0.16)	1.35***	(0.16)	1.35***	(0.16)	1.35***	(0.16)
	two or more			1.89***	(0.23)	1.96***	(0.23)	1.85***	(0.23)	1.85***	(0.23)	1.86***	(0.23)	1.86***	(0.23)
*Job absence due to disability*	no (ref.)														
	yes			2.03***	(0.22)	1.99***	(0.22)	2.00***	(0.22)	1.99***	(0.22)	1.99***	(0.22)	1.99***	(0.22)
*Childhood circumstances*	most advantaged (ref.)														
	advantaged			0.39	(0.31)			0.16	(0.31)	0.16	(0.31)	0.16	(0.31)	0.16	(0.31)
	neutral			1.06***	(0.29)			0.65*	(0.29)	0.65*	(0.29)	0.65*	(0.29)	0.65*	(0.29)
	disadvantaged			1.89***	(0.30)			1.31***	(0.30)	1.31***	(0.30)	1.30***	(0.30)	1.30***	(0.30)
	most disadvantaged			3.10***	(0.32)			2.44***	(0.32)	2.44***	(0.32)	2.44***	(0.32)	2.44***	(0.32)
*Labour market disadvantage*	none (ref.)														
	mild					1.74***	(0.16)	1.35***	(0.16)	1.35***	(0.16)	1.36***	(0.16)	1.36***	(0.16)
	moderate					2.60***	(0.21)	2.19***	(0.21)	2.19***	(0.21)	2.20***	(0.21)	2.20***	(0.21)
	severe					3.73***	(0.37)	3.29***	(0.38)	3.29***	(0.38)	3.30***	(0.38)	3.29***	(0.38)
	very severe					5.52***	(0.99)	5.07***	(0.98)	5.07***	(0.98)	5.09***	(0.98)	5.09***	(0.98)
*Compensation index*										-0.28*	(0.13)			-0.03	(0.11)
*Integration index*												-0.28***	(0.06)	-0.27***	(0.07)
*Random parameters*															
*Level 1: within country*		6.337***	(0.042)	6.179***	(0.041)	6.171***	(0.041)	6.134***	(0.041)	6.134***	(0.041)	6.134***	(0.041)	6.134***	(0.041)
*Level 2: between country*		2.376***	(0.470)	1.828**	(0.364)	2.145***	(0.425)	1.862**	(0.371)	1.616*	(0.323)	1.064	(0.218)	1.060	(0.217)
*Statistics*															
*R* ^*2*^ *1 (level 1)*				.0491		.0516		.0629		.0629		.0629		.0629	
*R* ^*2*^ *2 (level 2)*				.4076		.1847		.3857		.5374		.7992		.8007	
*Log likelihood*		-36540.49		-36256.03		-36243.45		-36174.69		-36172.88		-36167.59		-36167.54	
*AIC*		73086.98		72540.06		72514.90		72385.37		72383.75		72373.18		72375.09	
*BIC*		73108.95		72642.57		72617.41		72517.17		72522.87		72512.29		72521.53	

Note. *p < 0.05; **p < 0.01; ***p < 0.001.

## Results

### Sample description

The sample included slightly more women than men (5629 vs. 5552), and the mean age was 68 years at the time of the SHARELIFE interview. The average number of observations across countries was 860, with smallest number in Austria (411) and largest number in Belgium (1237). The majority left the labour market at age 60 or older (40 per cent), and only a minority of respondents reported any period of disability (21 per cent) or job absence due to disability (8 per cent). According to our definition of a critically elevated level of stressful work 16 per cent of the respondents were exposed. Considering childhood circumstances there was a rather high prevalence of adversity, with nearly half of the respondents reporting disadvantaged or very disadvantaged childhood circumstances. Conversely, labour market disadvantage was not frequent as the large majority (over 80 per cent) experienced mild or none disadvantage in their past careers (see Table 1 for details).

### Stressful work in European countries

Figure 1 displays country-specific percentages of stressful work and their geographical distribution across Europe. Prevalence is highest in Eastern and Southern Europe, whereas lowest rates are observed in the two Scandinavian countries, together with Switzerland and the Netherlands.

### Stressful work and the two proximal determinants

As displayed in Figure 2, childhood circumstances and labour market disadvantage are clearly related to the prevalence of stressful work. For both variables, we see a stepwise increase of percentage of stressful work with each level of adversity, with significant results in both cases (Childhood circumstances: chi^2^ (4) = 329.35, p < 0.001, labour market disadvantage: chi^2^ (4) = 164.47, p < 0.001).

### Stressful work and distal determinants (policy indices)

**Figure 3:**
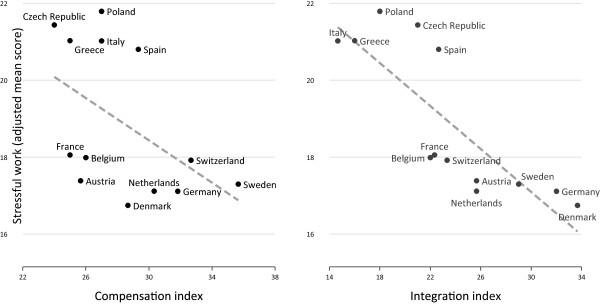
**Adjusted mean scores of stressful work among older men and women (N = 11181) and policy indices.**

How are the two macro indicators related to stressful work? Answers are given in Figure 3, where mean scores of stressful work (adjusted for country composition) are plotted against the two policy indices. In case of the compensation index, associations are slightly less pronounced as we observe a group of countries with low compensation scores (low levels of system generosity) and low mean level of stressful work (Austria, France, Belgium) (R^2^ = 24.2). In contrast, an almost linear association is observed in case of the integration index where more pronounced integration policies are related to lower mean scores of stressful work (R^2^ = 66.5).

### Results of multivariate analyses

Results of multilevel analyses testing our research hypotheses are presented in Table 2. The empty model shows significant variations both at the individual and at the country level, with an intra-class correlation (ICC) of 0.12. This indicates that 12 per cent of total variations in stressful work are due to differences between countries.

Turning to the individual predictors and the fixed parameters, findings show that men and those who had no disability during their life generally report less stressful work. The same holds true for older respondents and those retiring later. With regard to our main research questions and the first two models, we observe a stepwise increase of the regression coefficients according to level of disadvantage during childhood (Model 1; hypothesis 1) and level of labour market disadvantage (Model 2). These associations with stressful work are in line with the findings presented in Figure 2. Importantly, when combining these two groups of explanatory variables into one model (Model 3), the regression coefficients of childhood circumstances are generally attenuated, indicating that part of the association between disadvantaged childhood circumstances and stressful working conditions is due to labour market disadvantage (hypothesis 2). Turning to Models 4 and 5, where national policy indices are introduced as distal determinants, we observe a strongly significant regression coefficient in case of integration policies. In case of compensation policies the coefficient was weaker (significant at the 5% level). Coefficients of all individual predictors remain almost unchanged in these models, including the full model. Estimates of the random parameters displayed in Table 2 indicate that the variation of stressful work between countries (R^2^2: proportional reduction of variance explained at country level) is only moderately explained by country compositions (39 per cent when combining the two proximal determinants ‘childhood circumstances and ‘labour market disadvantage’ , Model 3). In contrast, the inclusion of one of the two distal determinants, the index of national integration policies, results in a non-significant standard deviation. In this latter model (Model 5) a proportion as high as 80 per cent of the between-country variation is explained (with a similar result in the Full Model) (hypothesis 3).

## Discussion

This contribution used data from 13 European countries based on the SHARE survey and analysed stressful work assessed retrospectively among older men and women (N = 11.193). Our first aim was to analyse associations between a proximal determinant, childhood circumstances, and stressful work. As a second aim, we studied the extent to which labour market disadvantage contributes to the explanation of this association. Third, we explored the effects of distal determinants in terms of national labour and social policies (compensation and integration policies) on stressful work.

With regard to the first hypothesis, we found strong support that adversity during childhood is related to stressful work. This is in line with previous research [5, 6, 8, 9, 43], but several aspects of our results deserve special attention. First, the retrospective assessment of stressful work in this study covered a time period that was more extensive than the one explored in other studies. Second, by studying this association across 13 European countries, we extended previous research evidence that was restricted to one or a few countries only. Third, with the results related to our second hypothesis we demonstrated an indirect effect of childhood circumstances on stressful work, partly mediated by disadvantaged labour market access. However, as the former association remained statistically significant after adjustment, it seems likely that this remaining direct effect reflects compromised coping abilities during childhood that aggravate the respondents’ vulnerability to chronic stress at work experienced later on [20, 44].

With regard to the third research question, we found that levels of stressful work were particularly high in Eastern and Southern countries, followed by Western Europe and lowest in Northern Europe. Notably, this pattern remained virtually unchanged after considering various factors of country composition. Concerning the third hypothesis on distal determinants of stressful work strong support was obvious in case of the summary index of national integration policies, but not in case of the second index, compensation policies. The association of well-developed national integration policies with mean levels of stressful work is in line with some previously reported results [12, 13, 16, 45]. However, our finding adds a new element: By distinguishing between policies related to employment protection (compensation index) and those describing established measures of employment activation (integration index), we used detailed evaluations of two specific types of interventions [17] that take into account a recent shift in emphasis from more passive to more active policies in research on welfare regimes in modern societies [18].

The finding that evidence in favour of our third hypothesis was limited to integration policies needs further consideration. It is possible that integration measures are more closely related to overall levels of stressful work than compensation policies, e.g. due to the fact that they target the needs of employed people who were exposed to precarious work at some stage of their career more closely. Nevertheless, potential protective effects of compensation policies should be explored in further studies. For instance, a recent study found that these latter policies to some extent may mitigate effects of work stress on mental health [46].

### Limitations

The following limitations must be considered. First, the data measuring core constructs, childhood circumstances, labour market disadvantage and stressful work, were collected retrospectively among older men and women who were retired at the time of data collection. This carries the risk of systematic reporting bias, where information may be positively tuned due to a tendency of harmonizing conflicting retrospective biographical accounts [47]. Yet, a high prevalence of disadvantaged childhood circumstances (Table 1) and levels of work stress comparable to those collected in samples of still employed people [45] do not support this argument. At the same time, the method of collecting retrospective information via the lifegrid approach was shown to provide accurate information in several areas of people’s life histories [26, 27, 29]. In a detailed study of SHARELIFE data a recent report found a rather convincing degree of accordance between interview data on childhood circumstances and data from official sources [27]. Obviously additional data allowing for bias control due to distinct personality characteristics or attribution styles would have been desirable, but was not available in this study. A second limitation concerns our choice of the two indicators of distal determinants of stressful work. These indicators may not cover relevant labour and social policies to a sufficient extent, and their measurement was rather crude, as information was taken from administrative data sources available from OECD. Certainly, these indices run the risk of bypassing more targeted national developments within single countries, and thus fail to do justice to a rich variation of political and socio-cultural traditions across Europe. Along this argument, the operational measures of the two indices of proximal determinants (childhood adversity and labour market disadvantage) can be criticized for their restricted comprehensiveness. Third, the number of countries included in this analysis was still relatively small when studying variations between countries within multilevel modelling. Extending the range of countries would increase the robustness of findings. Fourth, we must be cautious when interpreting the statistical significance of results in view of a large sample size. Yet, the consistency of findings and their fit with the theoretical framework support their validity. Finally, any generalization of findings needs to take into account the fact that we studied a distinct age cohort [48].

These limitations are balanced by several strengths. First, the SHARE study meets high quality standards of data collection, specifically a vigorously controlled study protocol, the application of validated questionnaires, the observation of standard procedures of translating the measures into different languages and of collecting and controlling the data [23]. Second, to our knowledge, this is the first study that combines the analysis of proximal and distal determinants of stressful work within a comprehensive study design that applies life history data in the context of a comparative cross-national survey. Third, given the foundation of our measurement of stressful work in established theoretical models of a health-adverse psychosocial work environment, results may point to relevant proximal and distal entry points of intervention measures that aim at reducing stressful work and improving working people’s health.

## Conclusion

This study demonstrates that distinct proximal (childhood circumstances and labour market disadvantage) and distal factors (national labour market policies supporting integration into paid work) are associated with levels of perceived stressful work during people’s occupational career. Our study illustrates the heuristic value of a broader analytical framework, as well as the promise of retrospective data, in analysing determinants of stressful work.

## Electronic supplementary material

Additional file 1: Table S1: Items measuring stressful work. **Table S2.** Policy indexes across countries. (DOC 54 KB)

## Abbreviations

AIC: Akaike information criterion
BIC: Bayesian information criterion
CAPI: Computer assisted personal interview
ICC: Intra-class correlation
ISCO: International standard classification of occupations
OECD: Organisation for economic co-operation and development
SHARE: Survey of health ageing and retirement in Europe.
