# Supplementary material for: Proximal and distal determinants of stressful work: framework and analysis of retrospective European data
Source: BMC Public Health. 2014 Aug 15;14:849. doi: 10.1186/1471-2458-14-849 (PMC4141949; doi:10.1186/1471-2458-14-849)
Supplement: Supplementary file 1 — Additional file 1: Table S1: Items measuring stressful work. Table S2. Policy indexes across countries. (DOC 54 KB) [file 12889_2014_6967_MOESM1_ESM.doc]

**Additional file 1**

Table S1. Items measuring stressful work

| **Measure** |  | **Item (strongly agree, agree, disagree, strongly disagree)** |
| --- | --- | --- |
|  |  |  |
| Work stress (main job) | Physical demands | 1. My job as [main job title] was physically demanding. |
|  |  | 1. My immediate work environment was uncomfortable (e.g. noise, heat, crowding). |
|  | Psychosocial demands | 1. I was under constant time pressure due to heavy workload. |
|  |  | 1. My work was emotionally demanding. |
|  |  | 1. I was exposed to recurrent conflicts and disturbances. |
|  | Social support at work | 1. I received adequate support in difficult situations. |
|  |  | 1. There was a good atmosphere between me and my colleagues. |
|  | Control | 1. I had very little freedom to decide how to do my work. |
|  |  | 1. I had an opportunity to develop new skills. |
|  | Reward | 1. I received the recognition I deserved for my work. |
|  |  | 1. Considering all my efforts and achievements, my salary was adequate. |
|  |  |  |
| Overall career evaluation |  | 1. All things considered, I am satisfied with my job career |
|  |  | 1. I experienced a major disappointment in my job career |
|  |  | 1. Considering all my efforts, I am satisfied with my work achievements. |
|  |  | 1. People close to me said I sacrificed too much for my job. |
|  |  | 1. My health has suffered from my job. |
|  |  |  |

Table S2: Policy indexes across countries

| **Compensation Index** |  | **Integration Index** |
| --- | --- | --- |
|  |  |  |
| 1. Population coverage (from total populations to employees only) |  | 1. Coverage consistency (access to different programmes and possibility to combine them) |
| 1. Minimum degree of incapacity needed for benefit entitlement |  | 1. Assessment structure (responsibility and consistency) |
| 1. Degree of incapacity needed for a full benefit |  | 1. Anti-discrimination legislation covering employer responsibility for work retention and accommodation |
| 1. Disability benefit level (in terms of replacement rate for average earnings with a continuous work record) |  | 1. Supported employment programme (extent, permanence and flexibility) |
| 1. Permanence of benefits (from strictly permanent to strictly temporary) |  | 1. Subsidised employment programme (extent, permanence and flexibility) |
| 1. Medical assessment (from exclusive responsibility of treating doctors to that of teams of insurance doctors) |  | 1. Sheltered employment sector (extent and transitory nature) |
| 1. Vocational assessment (from strict own occupation assessment to all jobs available) |  | 1. Vocational rehabilitation programme (obligation and extent of spending) |
| 1. Sickness benefit level (distinguishing short and long-term sickness absence) |  | 1. Timing of rehabilitation (from early intervention to late intervention only for disability benefit recipients) |
| 1. Sickness benefit duration (including the period of continued wage payment) |  | 1. Benefit suspension regulations (from considerable duration to nonexistent) |
| 1. Sickness monitoring (from no checks on sickness absence to strict steps for monitoring and early intervention) |  | 1. Additional work incentives (including possibilities to combine work and benefit receipt) |
|  |  |  |
| **Country (sumscore)** |  |  |
|  |  |  |
| Sweden (35.7), Denmark (28.7), Germany (31.8), Netherland (30.3), Belgium (26), France (25), Switzerland (32.7), Austria (25.7), Italy (27), Spain (29.3), Greece (25), Czech Republic (24), Poland (27), |  | Sweden (29), Denmark (33.7), Germany (32), Netherland (25.7), Belgium (22), France (22.3), Switzerland (23.3), Austria (25.7), Italy (14.7), Spain (22.7), Greece (16), Czech Republic (21), Poland (18) |
|  |  |  |

Note. Table is based on OECD (2003, 2010). Sumscores are average scores for 1985, 2000 and 2007.
